# Medical Department of the University of Louisville

**Published:** 1847-08

**Authors:** 


### MEDICAL DEPARTMENT OF THE UNIVERSITY OF LOUISVILLE.

The reader will not fail to note the circular of the Dean of the Medical Faculty of the University of Louisville, on the cover of the present number of our Journal. We understand that the gentlemen of the Faculty are looking forward to a large class in the ensuing autumn, and from the general prosperity of the country we see no reason why the medical schools generally should not attract good classes.

For the information of students, we will only add, that courses of lectures will be delivered in the months of September and October by Drs. Yandell, Cobb, Gross, and Bayless, on Chemistry, Anatomy, Surgery, and Obstetrics. These lectures will commence on the first Monday in September.

